# Citrus Extract High in Flavonoids Beneficially Alters Intestinal Metabolic Responses in Subjects with Features of Metabolic Syndrome †

**DOI:** 10.3390/foods12183413

**Published:** 2023-09-13

**Authors:** Mônica Maurer Sost, Yala Stevens, Bouke Salden, Freddy Troost, Ad Masclee, Koen Venema

**Affiliations:** 1 Centre for Healthy Eating & Food Innovation (HEFI), Maastricht University-Campus Venlo, 5928 SZ Venlo, The Netherlands; m.maurersost@maastrichtuniversity.nl; 2 BioActor BV, 6229 GS Maastricht, The Netherlands; yala.stevens@maastrichtuniversity.nl; 3 Department of Nutrition and Movement Sciences, School of Nutrition and Translational Research in Metabolism (NUTRIM), Maastricht University, 6200 MD Maastricht, The Netherlands; 4 Division of Gastroenterology-Hepatology, Department of Internal Medicine, Maastricht University Medical Centre, 6202 AZ Maastricht, The Netherlands; bouke.salden@mumc.nl (B.S.); a.masclee@mumc.nl (A.M.); 5 Food Innovation and Health, Department of Human Biology, School of Nutrition and Translational Research in Metabolism (NUTRIM), Maastricht University, 6200 MD Maastricht, The Netherlands

**Keywords:** citrus extract, metabolic syndrome, short-chain fatty acids (SCFAs), in vitro model, TIM-2

## Abstract

The objective of this study was to investigate the effects of a citrus extract rich in citrus flavonoids on intestinal metabolic responses in subjects with features of metabolic syndrome, in an in vitro colon fermentation system (TIM-2) and fecal samples obtained from human subjects in an in vivo trial. In the TIM-2 system inoculated with fecal samples of volunteers with features of metabolic syndrome, continuous citrus extract supplementation (500 mg/day) resulted in increased cumulative short-chain fatty acid (SCFA) levels compared to the control condition, which was mainly due to increased production of butyrate, acetate, and valerate. In human volunteers, 12 weeks of daily supplementation with 500 mg citrus extract resulted in a significant shift in the SCFA profile towards more butyrate (*p* = 0.022) compared to the placebo group. Furthermore, there was a trend towards a reduction in fecal calprotectin levels, a marker for intestinal inflammation, compared to the placebo (*p* = 0.058). Together, these results suggest that citrus extract intake may have a positive effect on intestinal metabolic responses and through this, on host health in subjects with features of metabolic syndrome. Further research is needed to provide more insight into the potential underlying mechanisms and to study effects on clinical parameters.

## 1. Introduction

Over the last decades, the Western diet and lifestyle have been emerging and spreading globally. This poses a significant issue for public health, as this lifestyle has been implicated in the development of various metabolic disorders, such as obesity, type 2 diabetes, and metabolic syndrome (MetS) [1,2].

In addition to diet and lifestyle-related factors, the composition of the intestinal microbiota should also be considered as a factor that can affect human metabolism and health [3]. Under physiological conditions, the intestinal microbiota can have a positive influence on host health by protecting against pathogen colonization, interacting with the intestinal epithelium and immune system and by providing energy and essential vitamins [4,5].

In the large intestine, dietary fibers are fermented by the microbiota, resulting in the production of short-chain fatty acids (SCFAs), consisting predominantly of acetate, propionate, and butyrate [6]. These microbial products with their diverse features, including anti-inflammatory effects and modulation of glucose and lipid metabolism [7], are valuable markers in research concerning both intestinal health and metabolic disorders. Furthermore, SCFAs can signal to receptors involved in the control of appetite-inhibiting hormones, such as peptide YY (PYY) and glucagon-like peptide 1 (GLP1), and may thereby be able to affect food intake [8]. In the skeletal muscle, acetate and butyrate might influence muscle fat oxidation and glucose metabolism via the activation of enzymes and receptor mechanisms [9]. However, disturbances in microbiota composition and microbial metabolism have been associated with metabolic conditions including obesity and MetS [10,11].

Data from previous studies [12,13,14] suggest that flavonoids present in citrus fruits, mainly hesperidin and naringin, can interact with the intestinal microbiota and can thereby beneficially affect factors related to metabolic and intestinal health. Intestinal microbes break down citrus flavonoids into smaller bioactive compounds that can be absorbed, while hesperidin and naringin can modulate the composition of the microbiota and affect SCFA production [15]. In vitro, an increase in the relative abundance of the *Clostridium coccoides*/*Eubacterium rectale* cluster was found after three weeks of treatment with 500 mg citrus extract that contained hesperidin-2S and naringin in the simulator of the human intestinal microbial ecosystem (SHIME). This change was accompanied by an increase in butyrate and total SCFA levels [13]. In a recent experiment using the dynamic TNO in vitro model of the colon (TIM-2; [16]) with fecal inocula obtained from healthy individuals, dose-dependent increases in *Roseburia*, *Eubacterium ramulus*, and *Bacteroides eggerthii* were found after treatment with citrus extract at doses of 250 and 350 mg/day for three days [12]. In addition, there was a shift in SCFA production towards more acetate production. In healthy volunteers, shifts in fecal microbiota composition and SCFA profiles have also been shown after treatment with orange juice (which contains citrus flavonoids in addition to other potentially bioactive substances), such as an increase in *Lactobacillus* spp. and in acetate levels relative to total SCFAs [14]. Based on these results, citrus flavonoids may have the potential to be used in novel preventive or therapeutic strategies targeting chronic diseases such as MetS.

Until now, data about the effects of citrus flavonoids on intestinal responses in metabolic health of human subjects and in particular those with metabolic disturbances, have been scarce. This study hypothesized that the flavonoids found in citrus fruits have the potential to interact with the intestinal microbiota and their metabolite production, thereby exerting positive effects on factors related to metabolic and intestinal health. Therefore, our objective was to investigate the effect of supplementation with a citrus extract rich in citrus flavonoids on intestinal metabolic responses in subjects with features of MetS. We studied these parameters both in an in vitro system (TIM-2) as well as in fecal samples of human subjects after citrus extract interventions.

## 2. Materials and Methods

### 2.1. In Vitro TIM-2 Study

In summary, the TIM-2 replicates the physiological conditions of the human proximal colon. This model consists of glass units with flexible walls that simulate peristaltic movements. These movements are induced by intermittent infusion of body-temperature water (37 °C) between the glass and the flexible walls, promoting mixing and transportation of luminal content. The pH is continuously monitored and maintained at 5.8, typical of the proximal colon. A dialysis system with semipermeable fibers allows the continuous removal of water and fermentation products, maintaining physiological concentrations of small molecules and electrolytes. A level sensor regulates luminal volume. To maintain an anaerobic environment, suitable for the growth of a microbiota similar to that of the human colon, gaseous nitrogen is flushed into the system continuously. All parameters are computer-controlled, and experiments were carried out in duplicate to ensure reproducibility of results [16].

#### 2.1.1. Collection and Preparation of Fecal Samples

Fresh fecal samples were collected from volunteers (*n* = 7; age 64 ± 6 years; 5 male, 2 female) with at least two of the following features for MetS: increased waist circumference (≥102 cm (males) or ≥88 cm (females)), increased serum triglycerides (≥1.7 mmol/L), reduced blood HDL-cholesterol (<1.0 mmol/L (males) or <1.3 mmol/L (females)), elevated blood pressure (systolic ≥ 130 mmHg and/or diastolic ≥ 85 mmHg), elevated fasting glucose (≥6.1 mmol/L) [17]. Volunteers with at least two diagnostic criteria of MetS were chosen, as we wanted to study the effects of citrus extract treatment in subjects at risk of MetS or those with relatively mild metabolic disturbances.

We homogenized the samples obtained from seven individuals, all conducted under anaerobic conditions, following the methodology described by Aguirre et al. [18] to establish a uniform microbiota pool. Subsequently, the resulting fecal slurry was rapidly frozen using liquid nitrogen and then carefully preserved at a temperature of −80 °C until the start of the experimental investigations.

#### 2.1.2. Study Product

The study product (MicrobiomeX^®^, BioActor BV, Maastricht, The Netherlands) was a commercially available citrus extract, with a standardized hesperidin-2S (>80%) and naringin (>4%) content. Both hesperidin and naringin possess properties that may translate into potential health benefits, such as anti-inflammatory effects, anti-oxidative effects, and the ability to interact with the intestinal microbiota [15,19]. Furthermore, changes in microbiota composition and SCFA profiles have been shown in previous studies using the same or similar citrus extracts [12,13]. Therefore, we chose this specific product for our study. The citrus extract was incorporated into the standard ileal efflux medium (SIEM) at a dosage of 500 mg/day. SIEM simulates the food residue remaining after passing through the ileocecal valve in the human small intestine that reaches the colon. The composition of SIEM consisted of starch, pectin, xylan, arabinogalactan, amylopectin, protein, vitamins, salts, Tween 80, and ox bile, as described previously [20,21]. Two TIM-2 units with SIEM only served as a control.

#### 2.1.3. Experimental Set-Up

The TIM-2 system was set up as described previously in detail by Sost et al. [12]. Briefly, each TIM-2 unit was inoculated with 30 mL of the standardized microbiota pool and 30 mL of pre-reduced dialysate. Subsequently, SIEM was gradually added to each unit during an adaptation period of 16 h (2.5 mL/h) followed by a 2 h starvation period. Following this, the supplementation was administered to the units for a duration of 72 h, utilizing a constant flow rate of 2.5 mL/h. The supplementation included either the SIEM alone or SIEM supplemented with 500 mg of citrus extract per day. Lumen and dialysate samples were obtained at baseline (t = 0), after 24, 48 and 72 h of treatment.

#### 2.1.4. Measurement of Short-Chain Fatty Acids and Branched-Chain Fatty Acids

SCFA and branched-chain fatty acid (BCFA) production was analyzed via GC-MS, as described by Nuenen et al. [22]. In brief, we centrifuged TIM-2 lumen samples for 10 min at 14,000 rpm. Then, 150 µL (lumen) of the clear supernatant was transferred to a glass-GC-vial containing 550 µL of the internal standard (a mixture of demineralized water, methanol, 2 mg/mL 2-ethyl butyric acid, and formic acid (20%)). The internal standard guarantees an accurate quantification and minimizes variability of the results. For TIM-2 dialysate samples, 300 µL dialysate was transferred to a glass-GC-vial containing 400 µL of a mixture of the internal standard. Of this mixture 0.5μL was loaded onto a DB-FATWAX Ultra Inert column (30 m, 0.25 mm, 0.25 μm; Agilent, Amstelveen, the Netherlands) in an Agilent 8890 GC System Custom, coupled to an Inert Plus MSD Turbo EI/CI, using an automatic sampler (PAL3 RSI 85, Agilent). We set the temperature settings of the injector port, oven, flame-ionization detector, and mass spectrometer detector to 250 °C, 200 °C, 275 °C, and 225 °C, respectively. Additionally, we maintained a carrier gas flow rate of 1.2 mL/min over the column. Concentrations were determined based on a calibration curve. Total SCFAs refers to the cumulative amount of acetate, propionate, butyrate, valerate, and caproate.

### 2.2. Clinical Study

The clinical study was part of a larger project investigating the effect of citrus extract on metabolic health [23]. The study was approved by the medical ethics committee of Maastricht University Medical Centre+ (MUMC+) and has been registered at ClinicalTrials.gov under NCT02610491. All participants signed informed consent before participation.

#### 2.2.1. Participants

Male and female adult volunteers (aged 18–65 y) were recruited by advertisements in local newspapers and on notice boards. To be eligible to participate in the study, participants needed to have two diagnostic criteria of MetS. Exclusion criteria included: diabetes mellitus (fasting plasma glucose of ≥ 7 mmol/L); any medical condition that could interfere with the study outcomes; smoking; excessive alcohol consumption (>20 alcoholic units/week); use of recreational drugs; recent changes in body weight (>3 kg weight gain or loss in previous three months); plans to lose weight or to change dietary habits during the study period; use of medication possibly influencing study endpoints; supplement use; pregnancy and lactation; allergy to test product or citrus fruits. These exclusion criteria were chosen as these factors could possibly influence the outcomes of the study or limit participation in the study.

The required sample size was calculated for the primary outcome of the original research protocol [23], which was glucose regulation assessed by OGTT (not included in this paper). Based on a previous study [24], a sample size of at least 50 subjects was required.

#### 2.2.2. Study Design and Protocol

The study was designed as a randomized, double-blind, placebo-controlled, parallel study with a duration of 12 weeks. Participants were randomly assigned to one of the two intervention groups: citrus extract or placebo (cellulose). The randomization was performed by an independent person using a computerized procedure. This researcher, who was not involved in any of the other study procedures, prepared the study products and was the only person with access to the randomization list containing the treatment allocation. All participants and researchers remained blind to treatment until all analyses had been completed. To assess compliance, participants were asked to keep all blister packs and any remaining capsules and bring them to the study site during the last visit.

During the entire study period, participants were asked to maintain their habitual diet. From four days before the start of the study until the last test day, participants were instructed not to consume foods high in citrus flavonoids (e.g., oranges, lemons, and grapefruit). Furthermore, participants were requested to abstain from vigorous physical exercise and consuming alcohol on the day before each test day. Anthropometric (height, weight, waist, and hip circumference) measurements were performed at baseline, after 6 weeks, and after 12 weeks of intake.

#### 2.2.3. Measurement of Fecal Calprotectin

Fecal samples were collected at home and stored at −20 °C until they were handed in at the test site. Fecal calprotectin concentrations were determined at baseline and after 12 weeks of intervention via a commercially available ELISA (Hycult Biotechnology, Uden, the Netherlands). The analyses were performed according to the manufacturer’s instructions. Before the analysis, samples were extracted by diluting aliquots of 100 mg of feces in 5 mL of extraction buffer (consisting of 0.1 M Tris, 0.15 M NaCl, 1.0 M Urea, 10 mM CaCl_2_.H_2_O, 0.1 M citric acid and 0.5% BSA, and with pH 8.0). This suspension was then vortexed, placed on a rotating shaker for 30 min at 4 °C, and centrifuged at 10.000 rpm for 20 min at the same temperature. The supernatant was aliquoted and stored at −20 °C until further analysis.

#### 2.2.4. Measurement of Short-Chain Fatty Acids and Branched-Chain Fatty Acids

Fecal SCFA and BCFA concentrations were measured at baseline and after 12 weeks of supplementation. For this analysis, approximately 1 g aliquots of fecal samples were processed by diluting and homogenizing them with 6 mL of demineralized water. Subsequently, centrifugation at 500× *g* was performed for 10 min to remove the particulate material, and resulting supernatants were then stored at −20 °C until further analysis [25]. The quantification of SCFA and BCFA concentrations in the supernatants was determined using gas chromatography with a flame ionization detector, as described previously by Possemiers et al. [26]. For the determination of total SCFAs, the same definition as described above was used (the sum of acetate, propionate, butyrate, valerate, and caproate).

#### 2.2.5. Analysis of Microbial Composition

*E. rectale*/*C. coccoides* was quantified in fecal samples collected at baseline and after 12 weeks. DNA extraction was performed according to the methods described by Boon et al. [27]. After that, a culture-independent qPCR method was used to quantify *E. rectale*/*C. coccoides*, as described previously [28]. Primer sequences were as follows: C. cocc-Fw: CGGTACCTGACTAAGAAGC; C. cocc-Rev: AGTTTYATTCTTGCGAACG.

#### 2.2.6. Study Product

For the clinical study, the same commercially available citrus extract (MicrobiomeX^®^, Maastricht, The Netherlands) as described above was used as the study product. Cellulose (500 mg microcrystalline cellulose; Aminolabs, Hasselt, Belgium) served as a placebo. Both the study product and placebo, were meticulously formulated into capsules, indistinguishable in both appearance and flavor, with each capsule containing 250 mg of the study product or the placebo. Two capsules were consumed each morning, just before breakfast with 200 mL water.

#### 2.2.7. Statistical Analyses

We performed the statistical analyses using IBM SPSS Statistics (version 27.0, IBM Corporation, Armonk, NY, USA). For numerical variables, we reported the baseline data as mean ± standard deviation (SD), while categorical variables were presented as numbers. Linear mixed model analyses were carried out using treatment, time, and treatment ∗ time as fixed factors. The data obtained with this model are presented as estimated mean ± standard error of the mean (SEM). A two-sided *p*-value of ≤ 0.05 was considered statistically significant.

## 3. Results

### 3.1. In Vitro TIM-2 Study

#### Production of Short-Chain Fatty Acids and Branched-Chain Fatty Acids

After 72 h of continuous citrus extract supplementation, the cumulative production of total SCFAs was higher compared to the control condition (SIEM only; Table 1). This was mainly due to the increased production of acetate, butyrate, and valerate. Production of the BCFAs, iso-butyrate and iso-valerate, was lower after 72 h of citrus extract supplementation compared to the control.

### 3.2. Clinical Study

In total, 91 participants were assessed for eligibility, of whom, 53 were randomized. Three participants dropped out before receiving the allocated intervention, due to personal reasons. All remaining 50 participants completed the study and were included in the analyses (Supplementary Figure S1). The baseline characteristics of the study participants are shown in Table 2. At baseline, participants were aged 51 ± 13 years and had an average body mass index (BMI) of 30.8 ± 3.8 kg/m^2^. In total, 18 male and 32 female participants completed the study.

#### 3.2.1. Anthropometric Outcomes

Anthropometric outcomes are shown in Table 3. BMI remained stable throughout the study period, with no differences between intervention groups. After 12 weeks of supplementation, there was a slight non-significant reduction (*p* = 0.087) in the waist-to-hip ratio in the citrus extract group compared to placebo.

#### 3.2.2. Calprotectin

**Figure 1:**
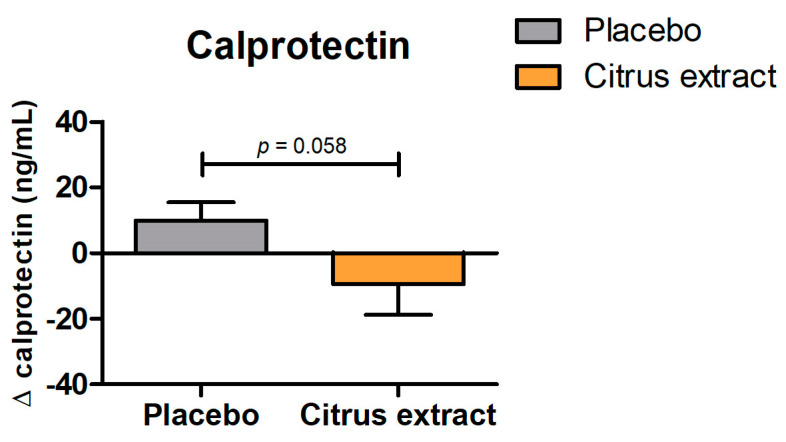
Changes in fecal calprotectin levels between baseline and after 12 weeks of placebo or citrus extract supplementation. All values are presented as mean ± SEM.

Fecal calprotectin is commonly used as a marker for intestinal inflammation. Changes in calprotectin levels over time are shown in Figure 1. After 12 weeks of daily supplementation with citrus extract, there was a trend towards a reduction in calprotectin levels, compared to placebo (*p* = 0.058; Figure 1).

#### 3.2.3. Short-Chain Fatty Acids and Branched-Chain Fatty Acids

**Figure 2:**
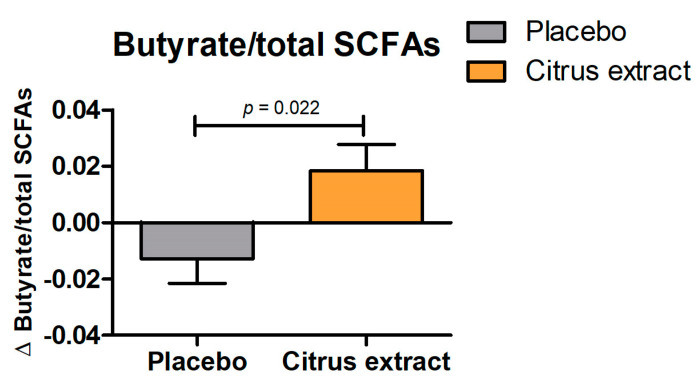
Changes in the butyrate to total short-chain fatty acids (SCFAs) ratio between baseline and after 12 weeks of placebo or citrus extract supplementation. All values are presented as mean ± SEM.

As shown in Table 4, fecal concentrations of total SCFAs, acetate, propionate, butyrate, valerate, caproate, and BCFAs were not significantly affected by 12 weeks of citrus extract intake (all *p* > 0.249). However, there was a significant increase in butyrate levels relative to the total SCFA concentrations in the citrus extract group compared to the placebo group (*p* = 0.022; Figure 2). Furthermore, there was an increase in the prevalence of butyrate-producing *E. rectale*/*C. coccoides* bacteria, but this effect was not significant (*p* = 0.383).

## 4. Discussion

Fermentative in vitro models serve as excellent substitutes for investigating the impact on intestinal microbiota function without some of the ethical constraints that are associated with human in vivo studies. In this context, the utilization of TIM-2, a validated, computer-controlled, and dynamic in vitro model of the colon [16], provided valuable insights into the effects of dietary compounds, such as flavonoids, for our clinical trial. According to Aguirre et al. [18] the use of a pool of microbiota for in vitro studies results in a bacterial community with a very similar profile and activity compared to those usually obtained from individual donors. Therefore, the use of pooled fecal samples from seven individuals was considered to be suitable for the fermentation experiments.

In this study, the impact of continuous supplementation with citrus extract on the production of SCFAs in TIM-2 was investigated. We found that after 72 h of supplementation, the cumulative production of total SCFAs was slightly higher compared to the control condition (SIEM). The increased SCFA levels were mainly accounted for by an increase in acetate, butyrate, and valerate production. This finding is consistent with previous studies that have reported increased SCFA production following supplementation with citrus extract high in the flavonoids, hesperidin and naringin [12,13].

We can most likely attribute the observed increase in SCFA production to the bioactive compounds present in citrus extract. These flavonoid compounds have shown growth-promoting effects on healthy intestinal bacteria, leading to the production of SCFAs [29]. Acetate, butyrate, and valerate are SCFAs that are widely recognized for their health benefits, including energy metabolism, gut health regulation, anti-inflammatory effects, and the suppression of cancer cells [30,31].

An increased level of acetate is expected to result in a more pronounced suppression of pathogenic microorganisms. In vitro studies have demonstrated an important role of SCFAs in the prevention of obesity-associated insulin resistance via various mechanisms [9]. In the skeletal muscle, acetate and butyrate increase muscle fat oxidation, possibly mediated via increased AMP-activated protein kinase (AMPK) activation and a peroxisome proliferator-activated receptors (PPAR) gamma-dependent mechanism. In addition, acetate and butyrate might influence skeletal muscle glucose metabolism in an AMPK-dependent manner, which may increase glucose uptake and glycogen storage [9]. In addition, the administration of acetate through lipid-based nanoparticles has been shown to effectively reduce ectopic lipid accumulation. This beneficial effect is achieved through the reduction of de novo lipogenesis in the liver and the suppression of lipolysis in adipose tissue [32].

Among the SCFAs, butyrate has received the most attention in scientific investigations. Multiple studies have consistently revealed it has positive effects due to its role as the primary energy source for colonocytes. Moreover, butyrate also plays a role in decreasing appetite and body weight by activating free fatty acid receptors (FFARs) in intestinal cells. This activation triggers the release of GLP-1 and PYY [33]. GLP-1 has been shown to boost insulin secretion and simultaneously suppress glucagon secretion, thereby regulating blood glucose levels. Conversely, PYY decreases appetite and decelerates gastric emptying, contributing to a reduction in food intake [34]. In addition, butyrate exhibits various beneficial effects on intestinal health. It not only stimulates the growth of intestinal epithelial cells but also enhances the integrity of the intestinal barrier while modulating the activities of intestinal microbiota and immune cells. A similar intestinal-barrier-function-promoting activity was demonstrated by valerate in an in vitro study [35]. These combined effects are crucial for maintaining a healthy intestinal barrier and its overall homeostasis. In TIM-2, we observed an increase in butyrate levels after treatment with citrus extract compared to control. This finding is in line with previous work, showing an increase in butyrate levels after three weeks of citrus extract treatment in an in vitro colon model that was inoculated with fecal samples from healthy volunteers [13]. In our clinical study, we also observed a shift in the SCFA profile towards more butyrate in the group supplemented with citrus extract. This finding suggests that the supplementation of citrus extract may impact the production of butyrate. Previously, an increase in the relative abundance of the butyrate-producing *E. rectale*/*C. coccoides* group was found after treatment with citrus extract in an in vitro model of the intestine [13]. Although we observed a non-significant increase in the prevalence of *E. rectale*/*C. coccoides* bacteria in the fecal samples of the participants, this trend supports the notion that citrus extract may have the potential to modulate the intestinal microbiota composition and promote the growth of bacteria associated with butyrate production. This is particularly relevant as certain microorganisms in the intestine can utilize both lactate and acetate as substrates for the synthesis of butyrate [6]. Together, these results add further support to the hypothesis that the flavonoids found in citrus fruits have the potential to interact with the intestinal microbiota and its metabolite production.

In addition to effects on appetite and the intestinal barrier, butyrate shows anti-inflammatory activity through its capacity of inhibiting the activation of pro-inflammatory signaling pathways in immune cells and by enhancing the expression of interleukin-10 [30]. These findings suggest that butyrate has the potential to mitigate inflammation. Chronic inflammation has been linked to the development and progression of metabolic disorders [36]. Therefore, using nutritional interventions to target inflammation may be a strategy to attenuate metabolic disease [37]. Several previous studies have shown that citrus flavonoids can exert anti-inflammatory effects in the intestine [15]. These effects may be due to an increase in butyrate levels, but in vitro evidence suggests that direct inhibition of inflammation by these flavonoids is also possible [38], indicating that it may be a combination of various factors. In line with these results, a trend towards a reduction in fecal calprotectin was observed in the clinical study after the intake of citrus extract. Although this effect did not reach statistical significance, this may be due to the fact that the sample size calculation for this study was based on a different outcome parameter and the study was not adequately powered to detect a difference in fecal calprotectin. These results at least point towards potential anti-inflammatory effects of citrus flavonoids in the intestine. Another limitation of this study relates to the methodology used to investigate the mechanisms related to the observed changes in SCFA production. The culture-independent qPCR method was limited in providing comprehensive insights into the interaction between the intestinal microbiota and its metabolic products. For a more comprehensive understanding of this interaction, future research should consider using more advanced techniques, such as 16S rDNA sequencing. Furthermore, in this study, subjects with only relatively mild metabolic disturbances were included. Lastly, some of the effects observed in this study concerned non-significant trends, suggesting that these results may have be to interpreted with caution.

In conclusion, this study showed that citrus extract treatment was able to induce a trend towards a more beneficial SCFA profile in a validated in vitro model of the colon and in human subjects. These results suggest that citrus extract intake may have a positive effect on intestinal metabolic responses and host health in subjects with features of MetS. To reaffirm our findings, future research with larger group sizes, involving populations with more pronounced metabolic disturbances is needed, such as individuals with MetS. The focus of such studies should be on elucidating the impact of citrus extract on intestinal metabolic response and establishing the underlying mechanisms of these effects.

## Figures and Tables

**Table 1:** Cumulative SCFA and BCFA production after 72 h of continuous supplementation with control (SIEM) or citrus extract.

	Control (SIEM)	Citrus Extract
Total SCFAs (mmol)	193.3	201.7
Acetate (mmol)	74.5	75.3
Propionate (mmol)	60.5	60.0
Butyrate (mmol)	53.4	61.2
Valerate (mmol)	2.8	4.2
Caproate (mmol)	2.1	1.0
BCFAs (mmol)	7.3	4.0
Butyrate/total SCFA ratio	0.28	0.30

SIEM, standard ileal efflux medium; SCFAs, short-chain fatty acids; BCFAs, branched-chain fatty acids.

**Table 2:** Baseline characteristics of the clinical study participants.

	Total Population (*n* = 50)	Placebo (*n* = 27)	Citrus Extract (*n* = 23)
Age, years (mean ± SD)	51 ± 13	50 ± 14	52 ± 11
Sex, Male/Female (n)	18/32	9/18	9/14
WHR (mean ± SD)	0.93 ± 0.07	0.91 ± 0.07	0.94 ± 0.07
BMI, kg/m^2^ (mean ± SD)	30.8 ± 3.8	31.4 ± 4.2	30.0 ± 3.2
BP systolic (mean ± SD)	131 ± 13	131 ± 17	131 ± 7
BP diastolic (mean ± SD)	82 ± 9	82 ± 10	83 ± 8

WHR, waist-to-hip ratio; BMI, body mass index; BP, blood pressure.

**Table 3:** BMI and WHR at baseline, after 6 weeks and after 12 weeks of placebo or citrus extract supplementation.

	Placebo (*n* = 27)			Citrus Extract (*n* = 23)			*P*1	*P*2
	Baseline	6 Weeks	12 Weeks	Baseline	6 Weeks	12 Weeks		
BMI (kg/m^2^)	31.4 ± 0.73	31.4 ± 0.74	31.5 ± 0.77	30.0 ± 0.79	30.0 ± 0.80	30.0 ± 0.83	0.616	0.514
WHR	0.91 ± 0.014	0.91 ± 0.013	0.92 ± 0.013	0.94 ± 0.015	0.94 ± 0.013	0.93 ± 0.014	0.617	0.087

All values are presented as estimated mean ± SEM. Differences between the placebo and citrus extract groups were compared via linear mixed model analyses with correction for baseline values. *P*1 and *P*2 represent the *p* values for the difference in estimated means between intervention groups after 6 and 12 weeks of intake, respectively. WHR, waist-to-hip-ratio; BMI, body mass index.

**Table 4:** SCFA and BCFA levels and butyrate-producing bacteria at baseline and after 12 weeks of placebo or citrus extract supplementation.

	Placebo (*n* = 27)		Citrus Extract (*n* = 23)		*p* Value
	Baseline	12 Weeks	Baseline	12 Weeks	
SCFAs and BCFAs					
Total SCFAs (µmol/g feces)	56.7 ± 5.0	65.0 ± 5.9	69.3 ± 5.5	75.4 ± 6.4	0.792
Acetate (µmol/g feces)	32.9 ± 3.0	38.4 ± 3.4	39.8 ± 3.3	42.9 ± 3.6	0.625
Propionate (µmol/g feces)	11.1 ± 1.3	12.9 ± 1.6	13.9 ± 1.4	14.1 ± 1.7	0.465
Butyrate (µmol/g feces)	10.4 ± 1.1	10.9 ± 1.3	12.4 ± 1.2	15.0 ± 1.4	0.249
Valerate (µmol/g feces)	1.8 ± 0.2	2.1 ± 0.2	2.3 ± 0.2	2.4 ± 0.2	0.602
Caproate (µmol/g feces)	0.47 ± 0.2	0.63 ± 0.2	0.89 ± 0.2	0.92 ± 0.2	0.651
BCFA (µmol/g feces)	3.1 ± 0.3	3.2 ± 0.4	3.6 ± 0.4	3.9 ± 0.4	0.807
Butyrate/ total SCFA ratio	0.18 ± 0.01	0.17 ± 0.01	0.18 ± 0.01	0.19 ± 0.01	0.022
Butyrate-producing bacteria					
*E. rectale/C. coccoides* (copies/g) × 10^10^	8.6 ± 1.3	9.5 ± 1.4	6.6 ± 1.4	9.5 ± 1.5	0.383

All values are presented as estimated mean ± SEM. Differences between the placebo and citrus extract groups were compared via linear mixed model analyses with correction for baseline values. SCFA and BCFA levels are expressed per gram fecal wet weight. SCFAs, short-chain fatty acids; BCFAs, branched-chain fatty acids.

## Data Availability

All raw sequence data and metadata are available from the corresponding author upon reasonable request.

## Supplementary Materials

The following supporting information can be downloaded at: https://www.mdpi.com/article/10.3390/foods12183413/s1, Figure S1. Consort flow chart.
