# Supplementary figures and images for: Citrus Extract High in Flavonoids Beneficially Alters Intestinal Metabolic Responses in Subjects with Features of Metabolic Syndrome
Source: Foods. 2023 Sep 13;12(18):3413. doi: 10.3390/foods12183413 (PMC10529306; doi:10.3390/foods12183413)

## Supplementary Materials

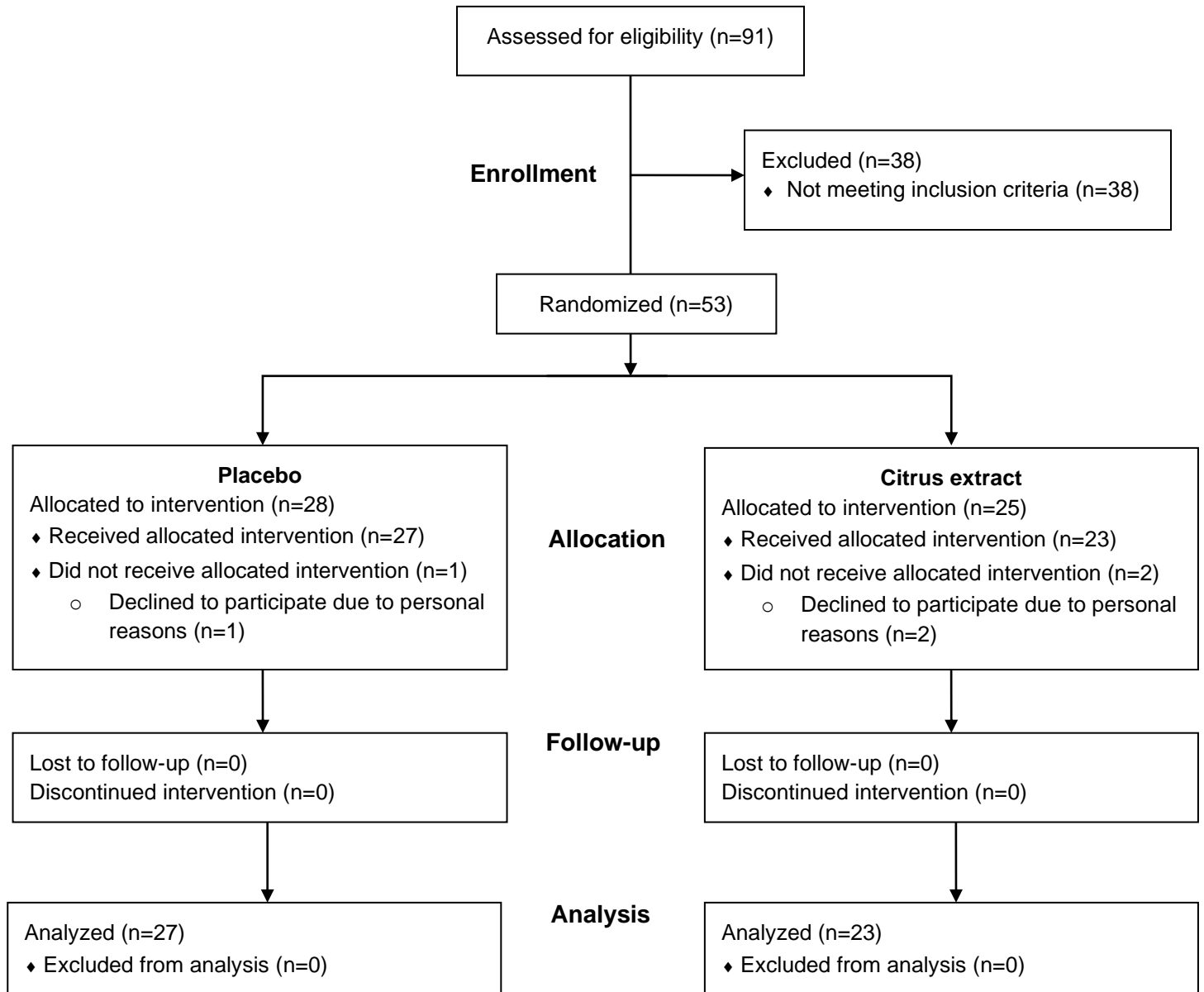

Figure S1. Consort flow chart

Supplement: Supplementary file 1 [file foods-12-03413-s001.zip › foods-2614437-supplementary.pdf]
